# Supplementary material for: Pathway profiling of a novel SRC inhibitor, AZD0424, in combination with MEK inhibitors for cancer treatment
Source: Mol Oncol. 2021 Dec 18;16(5):1072–90. doi: 10.1002/1878-0261.13151 (PMC8895456; doi:10.1002/1878-0261.13151)
Supplement: Supplementary file 1 — Fig S1. AZD0424 inhibits the activation of SRC in vivo. [file MOL2-16-1072-s005.pdf]

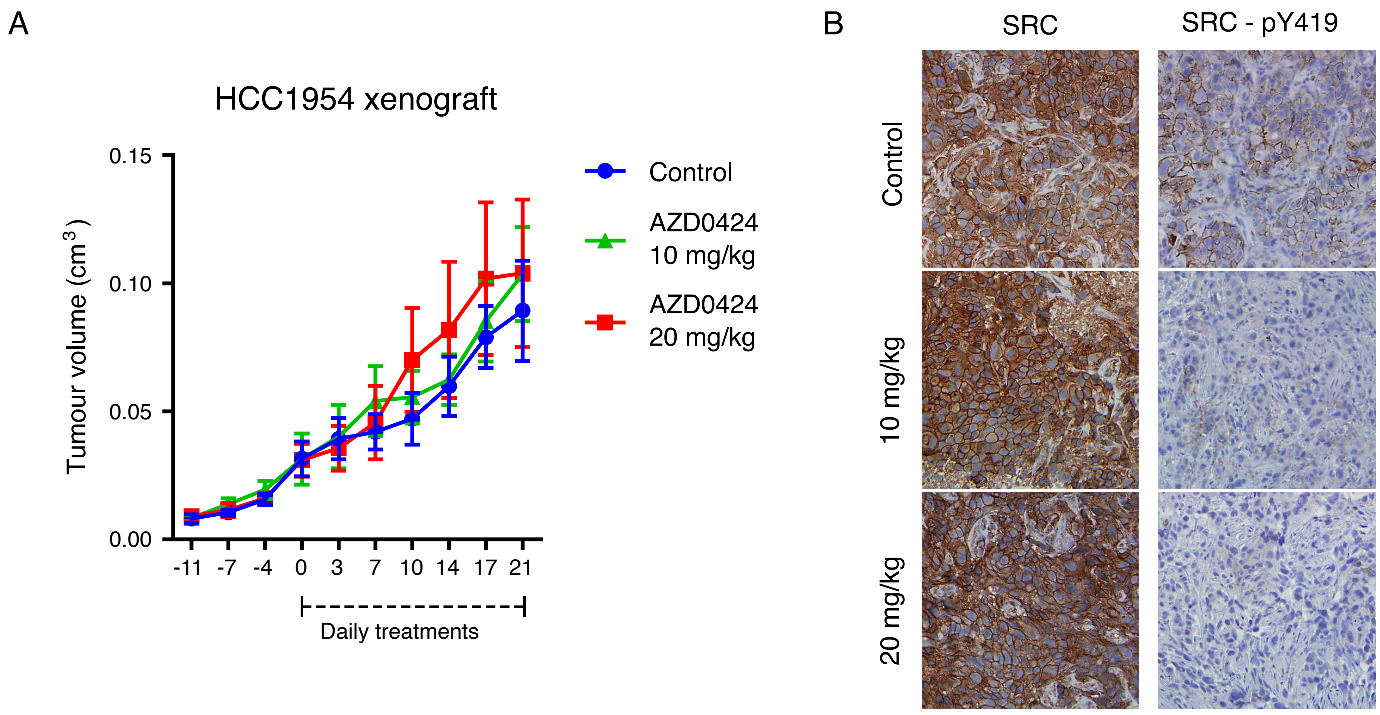

Supplementary Figure 1. AZD0424 inhibits the activation of SRC in vivo. (A), AZD0424 does not inhibit tumour growth of HCC1954 tumours. Tumour volumes are plotted as means ± SEM [n ≥ 4 mice per group (2 tumours per mouse)]. (B), Immunohistochemical analysis of phosphorylated SRC Tyr419 in HCC1954 tumours taken from mice treated with AZD0424.
