# Supplementary material for: Pathway profiling of a novel SRC inhibitor, AZD0424, in combination with MEK inhibitors for cancer treatment
Source: Mol Oncol. 2021 Dec 18;16(5):1072–90. doi: 10.1002/1878-0261.13151 (PMC8895456; doi:10.1002/1878-0261.13151)
Supplement: Supplementary file 3 — Fig S3. The combination of SRC and MEK inhibitors only weakly induces apoptosis in colorectal cell lines. [file MOL2-16-1072-s004.pdf]

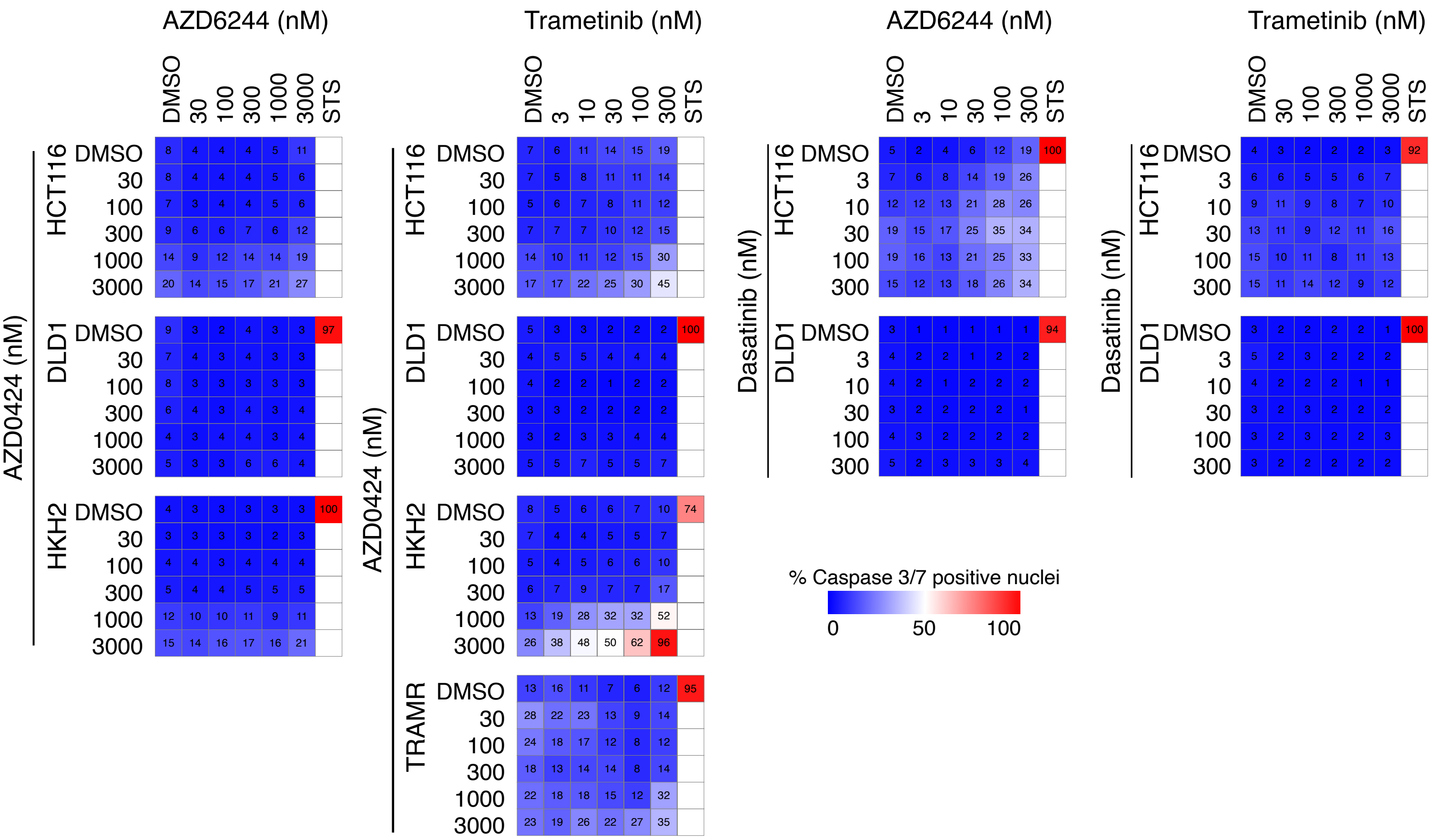

Supplementary Figure 3. The combination of SRC and MEK inhibitors only weakly induces apoptosis in colorectal cell lines.Activation of caspase 3/7 in cells (HCT116, DLD1, HKH2 or TRAMR) following 48 hours treatment with SRC inhibitors (AZD0424 or dasatinib) with MEK inhibitors (AZD6244 or trametinib) in combination.
