# Supplementary material for: Pathway profiling of a novel SRC inhibitor, AZD0424, in combination with MEK inhibitors for cancer treatment
Source: Mol Oncol. 2021 Dec 18;16(5):1072–90. doi: 10.1002/1878-0261.13151 (PMC8895456; doi:10.1002/1878-0261.13151)
Supplement: Supplementary file 4 — Fig S4. MAPK signalling is overactivated in trametinib‐resistant cells. [file MOL2-16-1072-s006.pdf]

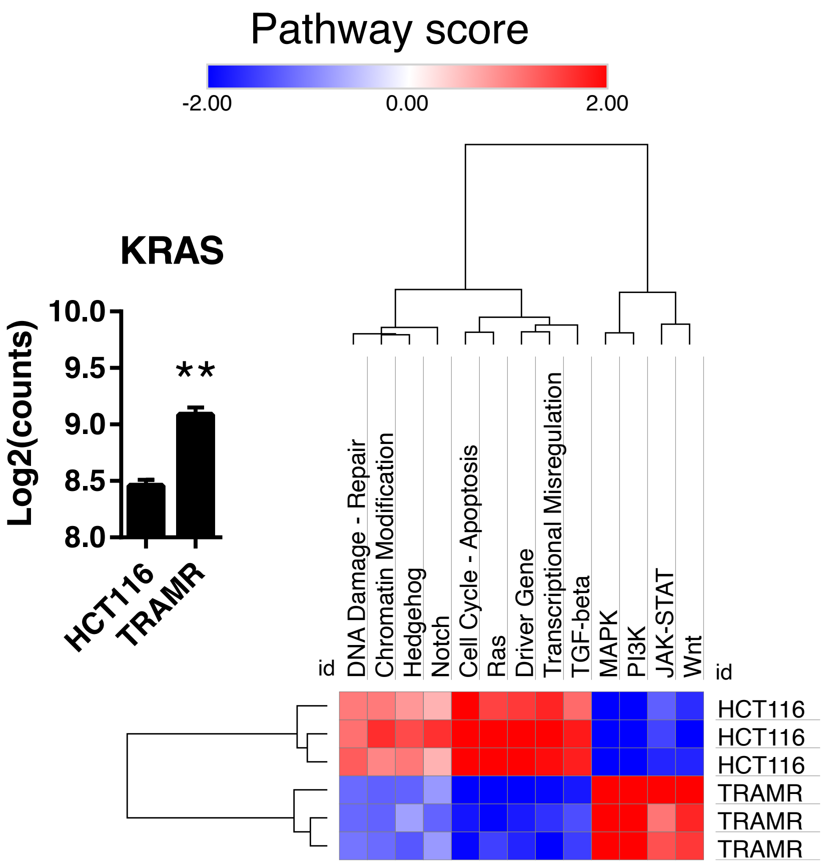

Supplementary Figure 4. MAPK signalling is overactivated in trametinib resistant cells. RNA from HCT116 and trametinib resistant TRAMR cells was analysed using a NanoString PanCancer Pathway panel. Inset bar chart shows the expression of KRAS gene. Mean cell expression is shown ± SEM (n = 3 independent experiments). **, $p < 0.01$ (t-test).
